# Genetic Causes of Non-pathogenic *Pseudomonas syringae* pv. *actinidiae* Isolates in Kiwifruit Orchards

**DOI:** 10.3389/fmicb.2021.650099

**Published:** 2021-03-25

**Authors:** Yue Li, Qiaomei Zhu, Taihui Zhi, Rong Fan, Ting Xie, Zhibo Zhao, Youhua Long, Zhong Li

**Affiliations:** ^1^Department of Plant Pathology, College of Agriculture, Guizhou University, Guiyang, China; ^2^Kiwifruit Engineering and Technology Research Center, Guizhou University, Guiyang, China

**Keywords:** non-pathogenic, bacterial canker of kiwifruit, transposable elements, comparative genomics, T3SS, *hrpR*

## Abstract

Bacterial canker disease has become the largest threat to kiwifruit cultivation and production. A monomorphic subpopulation of *Pseudomonas syringae* pv. *actinidiae* biovar 3 (Psa3) is responsible for the pandemic worldwide. Diversity in pathogenicity has been found in the pandemic subpopulation and in other Psa3 subpopulations causing epidemics in China. However, the genetic bases have not yet been elucidated. In this study, 117 Psa3 isolates were identified by Psa- and Psa3-specific primers, and evaluated for pathogenicity. Three isolates G4, G40, and S2 are not pathogenic to kiwifruit and do not elicit hypersensitivity responses (HRs) in non-host *Nicotiana benthamiana* leaves. Two isolates, G25 and G35, exhibited attenuated HR-eliciting activity in non-host *N. benthamiana*, but they exhibited greatly and slightly reduced pathogenicity in host plants, respectively. The genomes of the five isolates were sequenced and compared with closely related isolates revealed by MLVA and whole-genome typing methods. The candidate genetic loci responsible for the changes in pathogenicity and HR elicitation, were further evaluated by allele replacement experiments. We found that the three non-pathogenic isolates were formed due to the independent, identical insertion events of ISPsy36 transposon in the *hrpR* gene, encoding a key regulator of type III secretion system (T3SS) and type III effectors (T3Es). In the symptomatic sample from which G4 was isolated, 27% HR negative isolates were detected. In isolate G25, transposon insertion of ISPsy32 at the non-coding sequence upstream of the *hrpR* gene was detected, similar to a previously reported low-virulent Psa3 strain M227. In isolate G35, we detected disruptions of T3Es hopBB1-1 and hopBB1-2, which induce HR in *N. benthamiana* leaves revealed by *Agrobacterium tumefaciens* infiltration. These phenotype-changed isolates were formed at low frequencies during the course of pathogen infection in host plants, supported by the binding assay of ISPsy32 and the non-coding DNA sequences upstream of the *hrpR* gene, the co-isolation of the virulent isolates belonging to the same MLVA clade, and the low levels of transcription of the transposon genes. Taken together, in terms of short-term field evolution, transposon insertions in the T3SS-related genes resulted in the formation of non-pathogenic and low-virulent Psa3 isolates.

## Introduction

Kiwifruit (*Actinidia* spp.) is an economically important fruit plant cultivated in many countries, such as New Zealand, China, and Italy. However, bacterial canker disease, caused by *Pseudomonas syringae* pv. *actinidiae* (Psa) has spread across all the key kiwifruit production areas, and represents the largest threat to kiwifruit cultivation and production (Vanneste, 2017). Many efforts have been made to uncover the population structure of Psa, and at least five biovar populations (biovar 1, 2, 3, 5, and 6) within Psa has been identified, of which biovar 3 (Psa3) is responsible for the global pandemic (Vanneste et al., 2013; McCann et al., 2017; Sawada and Fujikawa, 2019). These biovars diverged many years ago and became distributed in different countries. For instance, the first recorded population, biovar 1, was detected in Japan, Italy, and Korea, and now is widely distributed in Japan; biovar 2 was only found in Korea, and biovars 5 and 6 occurred on a small scale in Japan (Sawada and Fujikawa, 2019).

Psa3 was thought to be the most aggressive biovar to kiwifruit, and remarkable differences in the composition of pathogenicity-related genes among these biovars have been observed (McCann et al., 2013). However, the molecular mechanisms underlying the emergence and rapid spread of Psa3 has not been fully elucidated. Psa3 was found in China in 1986 and has formed several distinct clonal-complexes related to geographical distribution (McCann et al., 2017; He et al., 2019; Zhao et al., 2019b). Notably, variations in pathogenicity were observed amongst strains within each genetically monomorphic clonal-complex, and the pathogenic determinants of Psa3 might be identified via a genomic comparison of phenotypically different strains within each clade (Zhao et al., 2019a). Similarly, the global clade within Psa3 which emerged in Italy in 2008, is genetically monomorphic (McCann et al., 2017), but an increase of virulence and genetic diversity of the Psa3 strains in Northern Italy has been described (Prencipe et al., 2018). Fitness increases of plant bacterial pathogen within host has been evidenced by experimental evolution as well (Guidot et al., 2014; Perrier et al., 2016). This supports the notion that Psa3 has evolved rapidly in agricultural ecosystems.

Genotypic diversity has been shown to arise within clonal populations during plant colonization by *P. syringae* (Barrett et al., 2011; Lovell et al., 2011; Bartoli et al., 2015). During the initial infection, non-pathogenic variants might benefit from co-existence with virulent pathogens (Barrett et al., 2011; Rufián et al., 2018). Non-pathogenic isolates may be the products of negative mutations during short-term evolution of the pathogen, and may be eliminated quickly due to their poor survivability in host plants. However, loss-of-function was also thought to be an adaptive strategy for pathogens and can be strongly selected *in planta* (Hottes et al., 2013; Perrier et al., 2016; Peyraud et al., 2016). For instance, the non-pathogenic variants in *Ralstonia solanacearum* populations exhibited better growth than their wild-type ancestor *in planta* (Peyraud et al., 2016; Perrier et al., 2019). The underlying genetic causes for the formation of non-pathogenic variants may provide clues for their biological significance. Meanwhile, non-pathogenic Psa3 isolates can be used for identification of pathogenic determinants of Psa3.

In this study, we obtained 117 Psa3 isolates from 15 kiwifruit orchards in China, and evaluated their pathogenicity in kiwifruit plants and their ability to elicit hypersensitivity responses (HRs) in non-host *Nicotiana benthamiana* leaves. The genetic causes of the non-pathogenic and reduced virulence isolates within Psa3 were identified by comparative genomics, and further evidenced by allele replacement experiments. The driving factor underlying the emergence of non-pathogenic variants, and the interaction between pathogenic and non-pathogenic variants within the host are discussed.

## Materials and Methods

### Plants, Bacterial Strains, and Growth Conditions

*N. benthamiana* plants and 3-years-old pot-grown plants of kiwifruit (*Actinidia chinensis* var. *chinensis* cultivar “Hongyang”) were cultivated in a phytotron at 24°C with a 12-h day/night cycle.

The Psa3 strains ICMP 18884 (Genome accessions CP011972.2, CP011973.1) and M227 (WGS accession MDXF01) were used in comparative genomic analyses. The 117 Psa3 isolates were identified from the symptomatic canes, leaves, and flowers of kiwifruit with bacterial canker disease as previously described (Zhao et al., 2013). The bacteria were grown in Luria–Bertani (LB) medium at 25°C for Psa strains, 28°C for *Agrobacterium tumefaciens* strains, and 37°C for *Escherichia coli* strains with appropriate antibiotics. The following concentrations (mg/L) of antibiotics were used: kanamycin, 50; nalidixic acid, 10; and ampicillin, 100. When necessary, Psa was cultured in HDM (*hrp*-derepressing medium) (Stauber et al., 2012), mimicing *in-planta* conditions, at 25°C.

### MLVA and Whole-Genome Typing for the Psa3 Isolates

The 117 Psa3 isolates were confirmed by PCR detection methods using the Psa-specific primers PsaF/R (Balestra et al., 2013) and Psa3-specific primers P0F/P6R (Gallelli et al., 2014). All the isolates were clustered by a previously established Multilocus Variable-Number of Tandem Repeats (VNTR) Analysis (MLVA) method (Zhao et al., 2019b). Briefly, 10 VNTR-related DNA products were PCR amplified for each isolate, and the repeat number in each locus was determined by capillary electrophoresis using a high-resolution automatic electrophoresis platform Qsep-1 (Bioptic Inc., Taiwan, China). The majority UPGMA tree was constructed by BioNumerics software (version 8.0; Applied Maths, St-Martens-Latem, Belgium) based on MLVA data for 117 Psa3 isolates. The genome tree was built based on the bulk of non-recombination SNPs from the genomic data of 57 representative Psa3 isolates, of which 14 were included in the MLVA analyses, using the panX pipeline (Ding et al., 2018).

### Pathogenicity Assays and Bacterial Infiltration in *N. benthamiana* Leaves

Pathogenicity tests of the Psa3 isolates were performed according to previously described methods: (1) wound inoculation on detached dormant kiwifruit canes with 10^8^ CFU/mL bacteria, (2) vacuum infiltration inoculation on leaf discs with 10^4^ CFU/mL bacteria (Zhao et al., 2019a). At least 15 canes or leaves were used in a pathogenicity trial, and the disease rating for each strain was calculated as a mean for three independent trials. Based on the pathogenicity data, a hierarchical cluster analysis was performed with the Ward’s method in SPSS 19.0. Analysis of variance (ANOVA) was performed to compare pathogenicity between strains using SPSS 19.0.

To test the ability to elicit HR in non-host plants, Psa3 isolates and mutants were cultured, suspended in 10 mM MgCl_2_ solution, and infiltrated into the upper leaves of 4-weeks-old *N. benthamiana* plants. The bacterial concentrations of the isolates used in the infiltration assay were designated in the figure legends.

*Agro*-infiltration assays were carried out following a previously described procedure (Zhao et al., 2019a). The *A. tumefaciens* strain GV3101 carrying an expression plasmid (pCAMIBA1300-effector) was cultured and infiltrated into *N. benthamiana* leaves. Cell death symptoms were evaluated and photographed after trypan blue staining 3–4 days post infiltration. Each assay was performed in triplicate.

### DNA Insertion and Mutant Generation

We performed comparative genomics as previously described (Zhao et al., 2019a). The candidate loci relating to pathogenicity were confirmed by allele replacement assays. The ISPsy36 transposon were naturally inserted at the 573-bp inside of *hrpR* gene, so we artificially inserted an 841-bp *gfp* fragment at the same location of the *hrpR* gene in the high-virulent Psa3 isolate G1, resulting in a mutant G1-*gfp*. The mutant was constructed using a previously described *SacB*-based unmarked mutagenesis method (Kvitko and Collmer, 2011; Zhao et al., 2019a). Briefly, a 2,499 bp fusion DNA fragment containing an 873 bp upstream flank (using primers hrpRN-F/R), an 841 bp *gfp* fragment amplified from pDSK-GFPuv (using primers gfpF/R) (Wang et al., 2007), and a 784 bp downstream flank (using primers hrpRC-F/R), was constructed by crossover-PCR and subsequently cloned into the *EcoR1/Hin*dIII sites on the suicide plasmid pK18*mobSacB*. Then, the fragment replaced the corresponding sequence in G1 as described in Zhao et al. (2019a), resulting in a mutant designated G1-*gfp*. The mutant was confirmed by PCR using hrpR_RTF/R primers and fluorescence using a NIKON CI-E + Ri2 fluorescence microscope (Nikon Instruments, Japan). The genes *hopBB1-1* (IYO_003727 in CP011972.2) and *hopBB1-2* (IYO_003675 in CP011972.2) were PCR amplified from G1 and inserted in the *Nde*I/*Pst*I locus of *Pseudomonas*-expressing plasmid pDSK-GFPuv (Wang et al., 2007), and the recombinant plasmids were electro-transformed into Psa3 isolate G35, resulting in overexpression mutants G35 pHopBB1-1 and G35 pHopBB1-2.

### qRT-PCR Analysis of Gene Expression

Bacteria were cultured in KB at the designated 0 h, and subsequently transferred into HDM medium with a final concentration of 0.02 OD_600_. The bacteria were sampled at the designated time and pretreated using the RNAprotect Bacteria Reagent (Qiagen). The total RNA was extracted with the RNeasy Protect Bacteria Mini kit (Qiagen). RNA integrity was evaluated by capillary electrophoresis with Qsep-1 (Bioptic Inc., Taiwan, China), and reverse transcription of the RNAs was performed with the RevertAid RT Reverse Transcription Kit (Thermo Fisher Scientific^TM^). Quantitative real time PCR was performed in a thermal cycler (CFX96, BioRad Inc., United States) using Fast SYBR^TM^ Green Master Mix (Applied Biosystems). Two house-keeping genes *gyrA* and *gyrB*, which constitutively expressed in bacteria, were used as the internal control in the RT-PCR examination. Primers used for qPCR are listed in Supplementary Table 1. Three replicates were performed for each sample. The relative expression levels of the *hrpL* gene of G1, G4, and G1-*gfp*, cultured in HDM, were analyzed using the 2^–ΔΔ^ ^CT^ method (Livak and Schmittgen, 2001).

### DNA Pull-Down Assay

This assay was carried out as described previously (Jutras et al., 2012). Briefly, the 1,076-bp non-coding DNA sequence upstream of the *hrpR* gene in Psa3 isolate G1 was synthesized and labeled by desthiobiotin at the 5′-end. The bioinylated DNA probe then bound to the Streptavidin-coated magnetic beads (Dynabeads M-280, Invitrogen), and the probe-bead complex was incubated with the cytoplasmic extracts of G1 cultivated in HDM. Finally, the probe-bead-protein complex was eluted and detected by SDS-PAGE. The binding proteins were identified by LC-MS/MS on a TripleTOF 5600 plus mass spectrometer (AB Sciex, Framingham, MA) coped with an ProteinPilot 5.0v software in Lianchuan Bio-company (Hangzhou, China).

## Results

### Diverse Clonal Populations Within Psa3 Spreading in China

**FIGURE 1:**
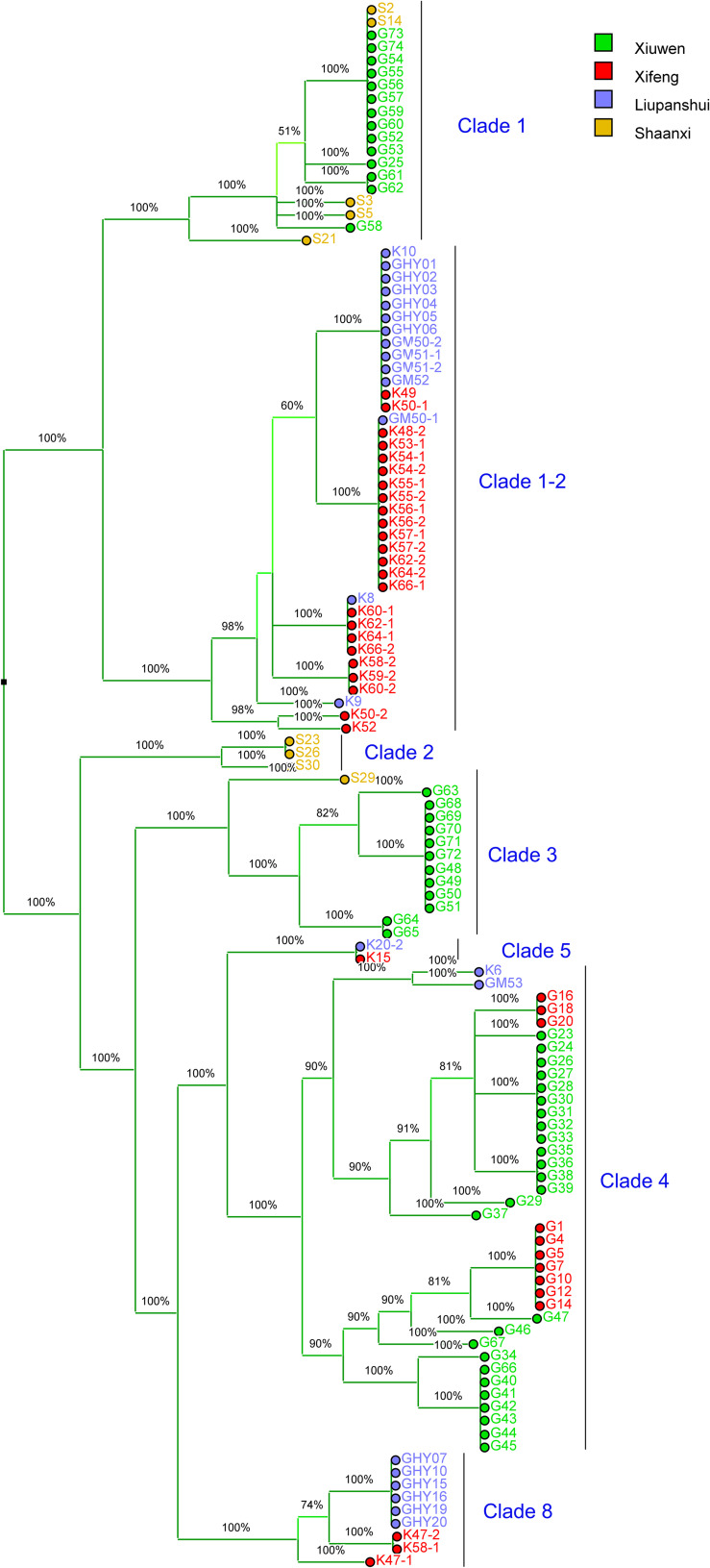
Majority UPGMA dendrogram constructed using Bionumerics 8.0 software based on MLVA data for 117 *Pseudomonas syringae* pv. *actinidiae* biovar 3 isolates from Guizhou and Shaanxi Province, China. The MLVA clustering method is as per Zhao et al. (2019b). The colored squares indicate the geographical source of the isolates. Xiuwen, Xifeng and Liupanshui are three main kiwifruit cultivating areas in Guizhou Province. The clade-populations are designated based on both MLVA typing results and whole-genome analysis.

**FIGURE 2:**
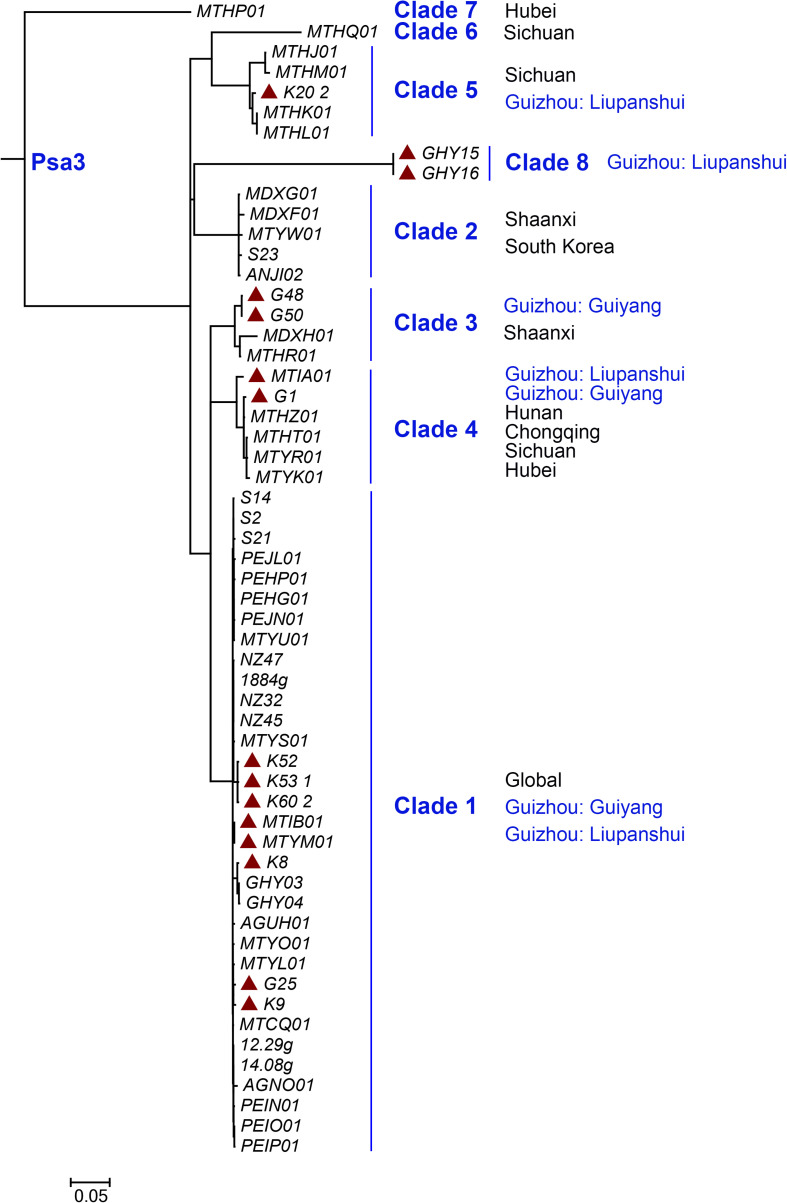
Maximum likelihood tree of 57 representative isolates within *Pseudomonas syringae* pv. *actinidiae* biovar 3 (Psa3) based on whole genome data. The tree was built based on the bulk of non-recombination SNPs from Psa3 genome data using the panX pipeline (Ding et al., 2018). The red solid triangle indicates Psa3 strains from Guizhou Province, China.

We identified 108 Psa3 isolates from an emerging kiwifruit cultivation region in China (Guizhou Province), and 9 Psa3 isolates from the largest kiwifruit cultivation region in the country (Shaanxi Province), in which the Psa3 population has been extensively investigated (Zhao et al., 2019a). All 117 isolates were confirmed as Psa3 by Psa- and Psa3-specific primers. We then evaluated the genetic diversity of Psa3 in the emerging region using a previously established MLVA method (Zhao et al., 2019b), and found 3–4 clonal populations in each area (Figure 1). The clustering result was further supported by whole-genome analysis (Figure 2). Seven clonal populations, which differed from each other with 200–300 non-recombinant SNPs at the genomic-wide level, have been described in China (McCann et al., 2017; Zhao et al., 2019a), and a novel clonal population, MLVA clade 8, was identified in this study. The clustering information was furthered used for the selection of reference strains in the subsequent comparative genomics between high and low (or non-) pathogenic isolates, and also for tracking the formation of the non-pathogenic isolates.

### Three Non-pathogenic and Two Virulence-Reduced Variants Identified

**FIGURE 3:**
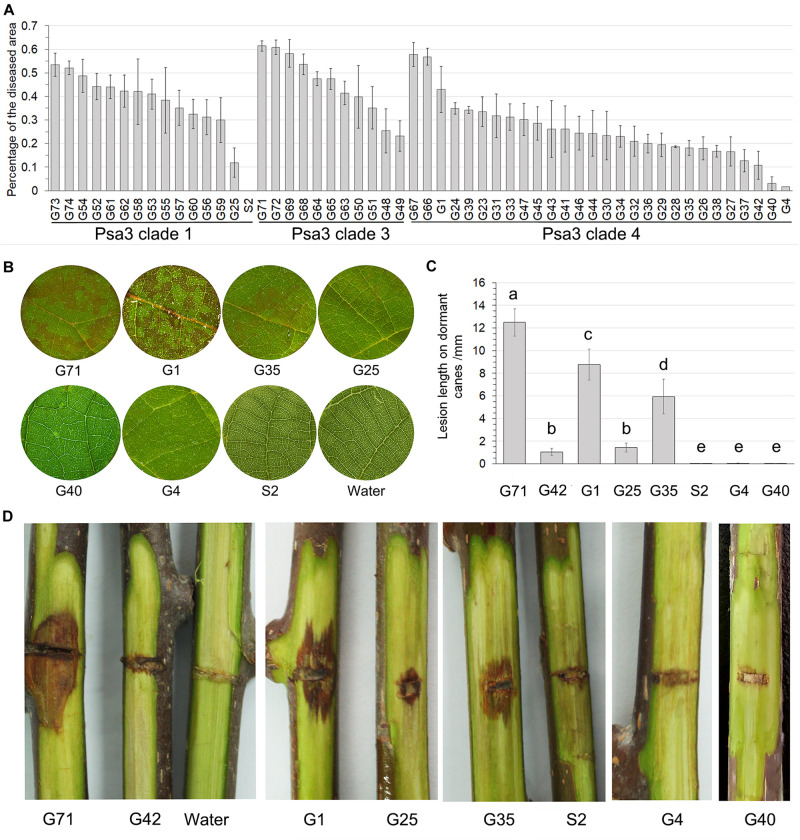
Non-pathogenic and very low-virulent isolates within *Pseudomonas syringae* pv. *actinidiae* biovar 3 (Psa3) were identified. **(A)** Diverse pathogenicity of the Psa3 isolates. The pathogenicity patterns of 54 Psa3 isolates from Guizhou Province were determined using a vacuum-infiltration inoculation method on leaf discs of *Actinidia chinensis* var. *chinensis* “Hongyang.” **(B)** Representative photographs of the non-pathogenic and very low-virulent isolates on kiwifruit leaf discs. **(C,D)** The pathogenicity of the non-pathogenic and very low-virulent isolates were confirmed by wound inoculation on detached dormant woody canes of kiwifruit cultivar “Hongyang.” Values are means ± standard deviations (SDs). The experiments were repeated three times with similar results.

**FIGURE 4:**
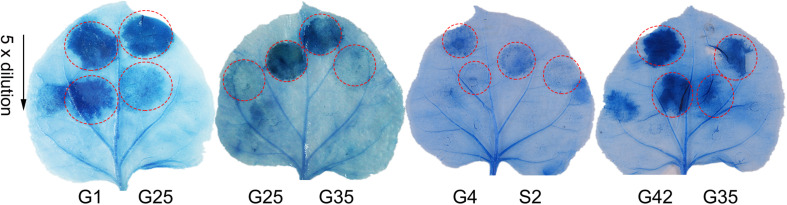
Non-pathogenic and very low-virulent isolates within *Pseudomonas syringae* pv. *actinidiae* biovar 3 (Psa3) showed attenuated capacity to elicit a hypersensitivity response (HR) in non-host *Nicotiana benthamina* leaves. Four zones in half a leaf were injected with fivefold diluted bacteria (10^7^ CFU/mL as the initial concentration), and three leaves from three independent plants were treated with each isolate. Photographs were taken 3 days post infiltration. Experiments were repeated at least twice with similar results.

The pathogenicity test of the 54 Psa3 isolates revealed variations in pathogenicity within each of the clonal populations (Figure 3A). Three isolates G4, G40, and S2 are not pathogenic, and two isolates G42 and G25 are less virulent to kiwifruit leaves (Figure 3B). The pathogenicity results were also evidenced on kiwifruit dormant canes using the wound inoculation method (Figures 3C,D). We further investigated the ability of the 117 isolates to elicit HR in non-host *N. benthamiana* leaves. None of the three non-pathogenic isolates showed any HR elicitation activity, while the two low-virulent isolates and another isolate G35 exhibited attenuated HR-eliciting activity in *N. benthamina* leaves (Figure 4). However, G35 showed moderate pathogenicity in host plants (Figure 3).

### Genetic Causes of the Phenotypically Changed Isolates

To uncover the genetic causes of the phenotypically changed isolates, comparative genomics were performed. The genomes of the six isolates were sequenced, and the reference high-virulent isolates were selected according to the MLVA clustering tree. Isolates S2 and G25, belonging to the Clade-1 population, were compared with the high-virulent strains ICMP 18884 (McCann et al., 2013) and M7 (Zhao et al., 2019a); isolates G4, G40, G42, and G35, belonging to the Clade-4 population, were compared with G1.

**FIGURE 5:**
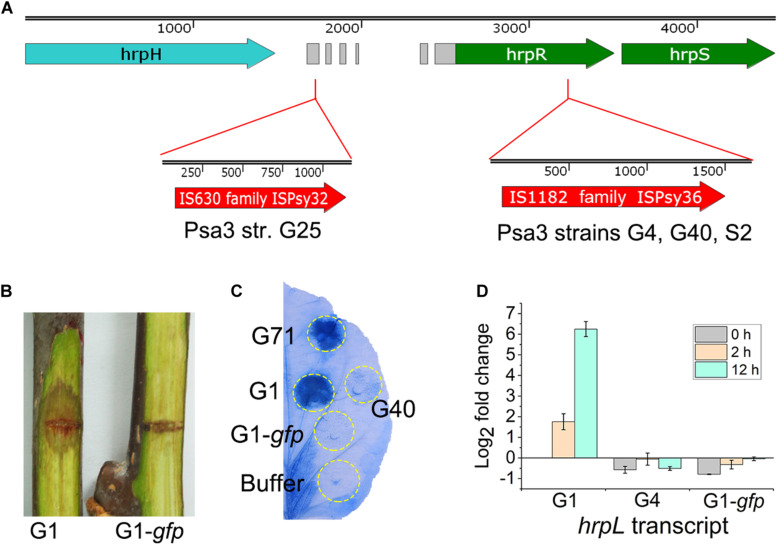
Genetic causes of the phenotypically-changed isolates within *Pseudomonas syringae* pv. *actinidiae* biovar 3 (Psa3). **(A)** ISPsy32 and ISPsy36 were inserted in the conserved region upstream and inside the *hrpR* gene, resulting in the low-virulent and non-pathogenic Psa3 isolates, respectively. The gray modules between the *hrpH* and *hrpR* genes represent the conserved sequences among diverse *P. syringae* pathovars. **(B,C)** An artificial mutant G1-*gfp* exhibited no pathogenicity on kiwifruit canes, and lost the ability to elicit a hypersensitivity response (HR) in non-host *Nicotiana benthamina* leaves. G1-*gfp* was constructed by an 841-bp *gfp* fragment insertion in the same location (as the ISPsy36 insertion in G25) of the *hrpR* gene in the high-virulent isolate G1. The *N. benthamina* leaves were infiltrated with bacterial suspensions (10^8^ CFU/mL) and photographed at 36-hour post infiltration. **(D)** The qRT-PCR results indicated that transcriptional patterns of the *hrpL* gene in G4 and G1-*gfp* were similar in the *hrp*-derepressing medium (HDM), while different from that of G1. Two house-keeping genes *gyrA* and *gyrB*, which constitutively expressed in bacteria, were used as the internal control. The experiments were repeated three times with similar results.

In the three non-pathogenic isolates (S2, G4, and G40), the insertion events of 1,666-bp ISPsy36 transposon (IS1182 family) were detected at the same position (573-bp) of the *hrpR* gene (Figure 5A), encoding the key regulator of type III secretion system (T3SS) and type III effectors (T3Es). We artificially inserted an 841-bp *gfp* fragment in the same location of the *hrpR* gene in G1, resulting in a mutant G1-*gfp*. The mutant exhibited no pathogenicity on kiwifruit canes (Figure 5B), and lost the ability to elicit HR in *N. benthamina* leaves (Figure 5C). Given the presence of the “HrpR/S-HrpL-T3SS/T3Es” hierarchical regulatory cascade in *P. syringae* (Xie et al., 2019), we detected the *hrpL* transcripts of the three strains in HDM, mimicking *in-planta* conditions. As expected, *hrpL* transcription in G4 and G1-*gfp* were no longer induced in HDM (Figure 5D). These results indicated that the transposon disruption of the *hrpR* gene is the cause of the phenotype of the above-mentioned non-pathogenic isolates. Two Italian Psa3 isolates, IPV-BO 8893 and IPV-BO 9286, were hitherto shown to be HR negative, but the genetic causes were not identified (Biondi et al., 2017). We reviewed the genomic data of the two HR negative isolates, and found transposon-insertion events at the same location in the *hrpR* gene.

In the low-virulent isolate G25, insertion of an 1,176-bp ISPsy32 transposon (IS630 family) was detected upstream of the *hrpR* gene (Figure 5A), identical to a previously reported low-virulent strain M227 (Zhao et al., 2019a). The insertion caused disorder expression of the *hrpR/S* gene and resulted in attenuated expression and secretion of T3SS and T3Es (Zhao et al., 2019a). However, we could not identify the genetic cause of G42, which is phenotypically very similar to G25.

**FIGURE 6:**
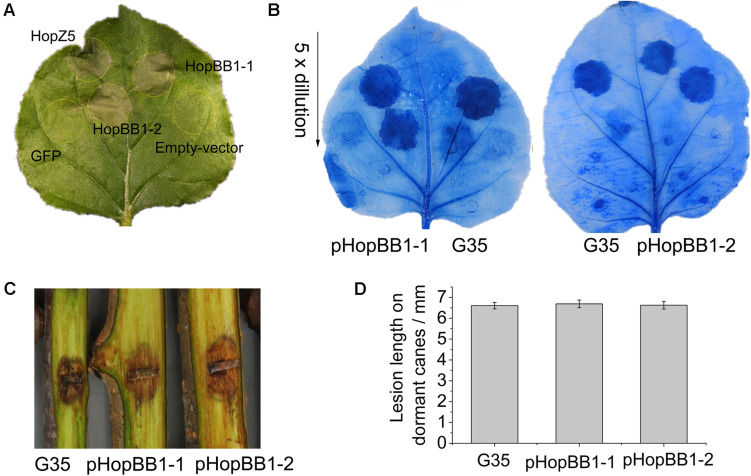
Type III effectors HopBB1-1 and HopBB1-2 induce hypersensitivity response (HR) in non-host *Nicotiana benthamina* leaves and enhance the HR-inducing ability of G35. **(A)** HR induction of heterologously expressed HopBB1-1 and HopBB1-2. The type III effector HopZ5 is used as the positive control, while the GFP and the empty-vector pCAMIBA1300 are used as the negative control. Four-weeks-old *N. benthamina* leaves were infiltrated with *Agrobacterium tumefaciens* strain GV3101 carrying certain plasmid at a concentration of 10^8^ CFU/mL, and were photographed at 36-hour post infiltration. The experiments were repeated three times with similar results. **(B)** Constitutively expressed HopBB1-1 or HopBB1-2 in G35 enhanced HR-inducing ability. **(C,D)** HopBB1-1 and HopBB1-2 constitutively expressed alone in G35 did not increase the pathogenicity of the pathogen on kiwifruit.

In the isolate G35, we found that two T3E genes, *hopBB1-1* and *hopBB1-2*, were incomplete. It has been reported that HopBB1-2 is localized in nucleocytoplasmic, and does not trigger HR in *N. benthamina* and *N. tabacum* Wisconsin 3 leaves (Choi et al., 2017). However, in this study we found that both HopBB1-1 and HopBB1-2 induced HR in *N. benthamiana* leaves (Figure 6A).Moreover, the G35 mutants expressing either *hopBB1-1* or *hopBB1-2* exhibited increased HR-eliciting activity (Figure 6B), but not pathogenicity (Figures 6C,D).

### Phenotypically-Changed Psa3 Isolates Formed During the Course of Pathogen Infection

We investigated whether the phenotypically-changed isolates were formed during the course of pathogen infection. In the symptomatic sample from which G4 was isolated, 27% HR negative isolates were detected, and both HR-negative and HR-positive isolates showed identical MLVA typing data and genomic sequences (Figure 1). The HR-negative isolate G40 and HR-positive isolate G41 were co-isolated and showed identical MLVA patterns (Figure 1). This indicated that the HR negative clones were formed during the course of infection, and could reach a high population level in host tissue.

The phenotypically-changed Psa3 isolates were caused by the potential active transposons. There are 48 complete ISPsy32 and 14 complete ISPsy36 transposons, respectively, presented in the Psa3 strain ICMP 18884 (Genome accession no. CP011972.2). We performed an *in vitro* DNA pull-down assay to detect the proteins binding to the upstream non-coding sequence of the *hrpR* gene in G1, and identified two transposon-associated proteins, ISPsy32 (IS630 family) and ISPsy34 (IS66 family) (Supplementary Table 3).

## Discussion

An important issue in plant-microbe co-evolution is how pathogens evolve within crop plants in natural conditions, but very few studies have investigated the trajectories of pathogen evolution (Arnold and Jackson, 2011). The emergence of Psa3 in China in the 1980s, concomitant with domestication and cultivation of kiwifruit, offers a rare opportunity to understand the short-term evolution of the pathogen and the responding evolutionary factors (McCann et al., 2017; Zhao et al., 2019b). In terms of the evolution of bacterial pathogenicity, we focused on the non-pathogenic and very low-virulent Psa3 isolates formed in Chinese kiwifruit orchards, and investigated the genetic causes of these isolates. Our results revealed that transposition events of the transposable elements (TEs) in the T3SS cluster were leading causes for these phenotypically-changed isolates.

### Independent, Identical TE Transposition Events Occurred During Pathogen Infection

The non-pathogenic Psa3 isolates were isolated in geographically different places, and were categorized into different MLVA clades (Figure 1). For instance, isolates G4 and G40 within the Clade-4 population, and S2 within Clade-1, were formed in two kiwifruit cultivated regions (Guizhou and Shaanxi Province), respectively (Figure 1). However, their changed phylotype and also a previously reported HR negative isolate in Italy (Firrao et al., 2018), were caused by identical transposition events of ISPsy36. Meanwhile, independent transposition events of ISPsy32 were found in the two low-virulent Psa3 isolates G25 and M227. The low-virulent G25 within Clade-1 and M227 within Clade-2 were isolated from Guizhou Province and Shaanxi Province, respectively (Figure 2). This is very interesting that the same mobile element was inserted at the exact same location in Psa3 strains isolated at different time and at different places. The target DNA sequences of ISPsy36 and ISPsy32 are “GGCC” and “CTAG” repeats, respectively. These two TEs and the corresponding targets are presented in all Psa pathovars, indicating the potential presence of phenotypically-changed isolates caused by TE insertions in other Psa pathovars.

These TEs are potentially active in Psa3, and the phenotype-changed isolates were formed during the course of pathogen infection in host plants. This was evidenced by an *in vitro* DNA pull-down assay, mimicking *in-planta* environments, and revealing that ISPsy32 indeed binds to the non-coding DNA sequences upstream of the *hrpR* gene in the T3SS cluster, which occurred in the formation of G25 and M227. Moreover, the co-isolation of the virulent and the phenotype-changed isolates, belonging to the same MLVA clade, further supported this view. However, the TE-associated genes showed low levels of transcription and were not upregulated during pathogen infection (McAtee et al., 2018), which accords with the observation that non-pathogenic isolates were only identified at low frequency during Psa3 isolation.

### The Fate or Biological Role of the Non-pathogenic Isolates

The non-pathogenic Psa3 variants might reach a high population level, together with the original high-virulent bacteria, in the initial infection, but might be eliminated due to natural selection. In a mixed infection, T3Es secreted by the virulent bacteria can favorably modify the host environment for the non-pathogenic variants, while the T3SS-deficient non-pathogenic strain may trigger the PTI (PAMP-triggered immunity)-based defense, impeding the spread of the virulent strain (Barrett et al., 2011; Rufián et al., 2018). Before now, the HR-negative isolates had never been identified alone in symptomatic samples. The non-pathogenic isolates may have decreased fitness in host plants.

However, it has been reported that less aggressive variants of *P. syringae* have a fitness advantage in non-host environments and are maintained in the field (Barrett et al., 2011). Thus, the non-pathogenic variants of Psa3 may play a role in adaptation to other stages of its life cycle.

The emergence of the non-pathogenic variants may also be interpreted by another scenario whereby a resource allocation trade-off mechanism exists in plant pathogenic bacteria (Peyraud et al., 2016; Perrier et al., 2019). For instance, the non-pathogenic variants of *R. solanacearum* were caused by a gene mutation of the quorum-sensing-dependent regulatory protein PhcA, and showed enhanced metabolic versatility compared to the wild-type (Perrier et al., 2019). Expression of T3SS represents a significant cost for Psa3, and the maintenance of a pathogenicity trait can be challenging. This would be verified by the discovery of the reversion of the natural non-pathogenic variant to the wild-type form after *in-planta* multiplication, as per that of *R. solanacearum* (Poussier et al., 2003).

### The Central Role of T3SS in Psa3 Pathogenicity

All the above results clearly support the central role of T3SS during the interaction of Psa3 with host and non-host plants. T3SS and T3E genes were induced in the early phase of Psa3 infection, and were required for pathogenicity (McAtee et al., 2018; Zhao et al., 2019a). It has been reported that Psa3 strain C17 naturally lacks the T3SS cluster and is highly compromised in its ability to grow in host plants (McCann et al., 2017). Moreover, a recent comparative transcriptional profiling exercise revealed that T3SS genes were strongly activated in Psa3 responding to apoplast-like conditions, compared with those in Psa1 and Psa2, which are responsible for the epidemics in Japan and Korea, respectively (Vandelle et al., 2020). Several T3Es secreted by the T3SS apparatus have been reported to be involved in pathogenicity in Psa3, such as HopZ5 (Zhao et al., 2019a), AvrE1, and HopR1 (Jayaraman et al., 2020). In this study, we found that T3Es HopBB1-1 and HopBB1-2 were incomplete in Psa3 isolate G35, which showed attenuated HR-eliciting activity in *N. benthamina* leaves and moderate pathogenicity in host plants. The two T3Es indeed induce HR in *N. benthamina* leaves, which evidenced by heterologous expression mediated by *A. tumefaciens*, and over-expression in G35. However, over-expression of HopBB1-1 or HopBB1-2 could not enhance the virulence of G35. The mobile DNA, including transposons, plasmids, or genomic islands (GIs) have been reported to be involved in T3E evolution, and genetic variation affecting T3E genes, and thus bacterial virulence and host specialization, can arise during interaction with the plant host (McCann and Guttman, 2008; Tampakaki et al., 2010; Arnold and Jackson, 2011; Karasov et al., 2014; Frantzeskakis et al., 2020). However, although there are remarkable differences in the composition of T3Es among different Psa biovars (McCann et al., 2013; Sawada and Fujikawa, 2019), only the T3SS apparatus and regulator, and not T3E genes, were involved in the rapid short-term evolution of Psa3 pathogenesis in kiwifruit orchards.

## Data Availability Statement

The datasets presented in this study can be found in online repositories. The names of the repository/repositories and accession number(s) can be found below: https://www.ncbi.nlm.nih.gov/bioproject, PRJNA693644 and http://www.proteomexchange.org/, PXD023722.

## Author Contributions

YuL and QZ carried out most of the experiments. TZ, TX, and RF conducted the data analysis and prepared the figures and tables. ZZ and YoL designed and supervised the experiments. ZZ and ZL wrote the manuscript. All authors reviewed and approved the manuscript.

## Conflict of Interest

The authors declare that the research was conducted in the absence of any commercial or financial relationships that could be construed as a potential conflict of interest.

## Supplementary Material

Supplementary Figure 1 Hierarchical cluster analysis of pathogenicity data with the Ward’s method in SPSS 19.0.

Supplementary Table 1 Primers used in this study.

Supplementary Table 2 The detailed information of Psa3 isolates and MLVA typing results.

Supplementary Table 3 Results of the DNA pull-down assay.
