# Supplementary figures and images for: Genetic Causes of Non-pathogenic Pseudomonas syringae pv. actinidiae Isolates in Kiwifruit Orchards
Source: Front Microbiol. 2021 Mar 25;12:650099. doi: 10.3389/fmicb.2021.650099 (PMC8027508; doi:10.3389/fmicb.2021.650099)

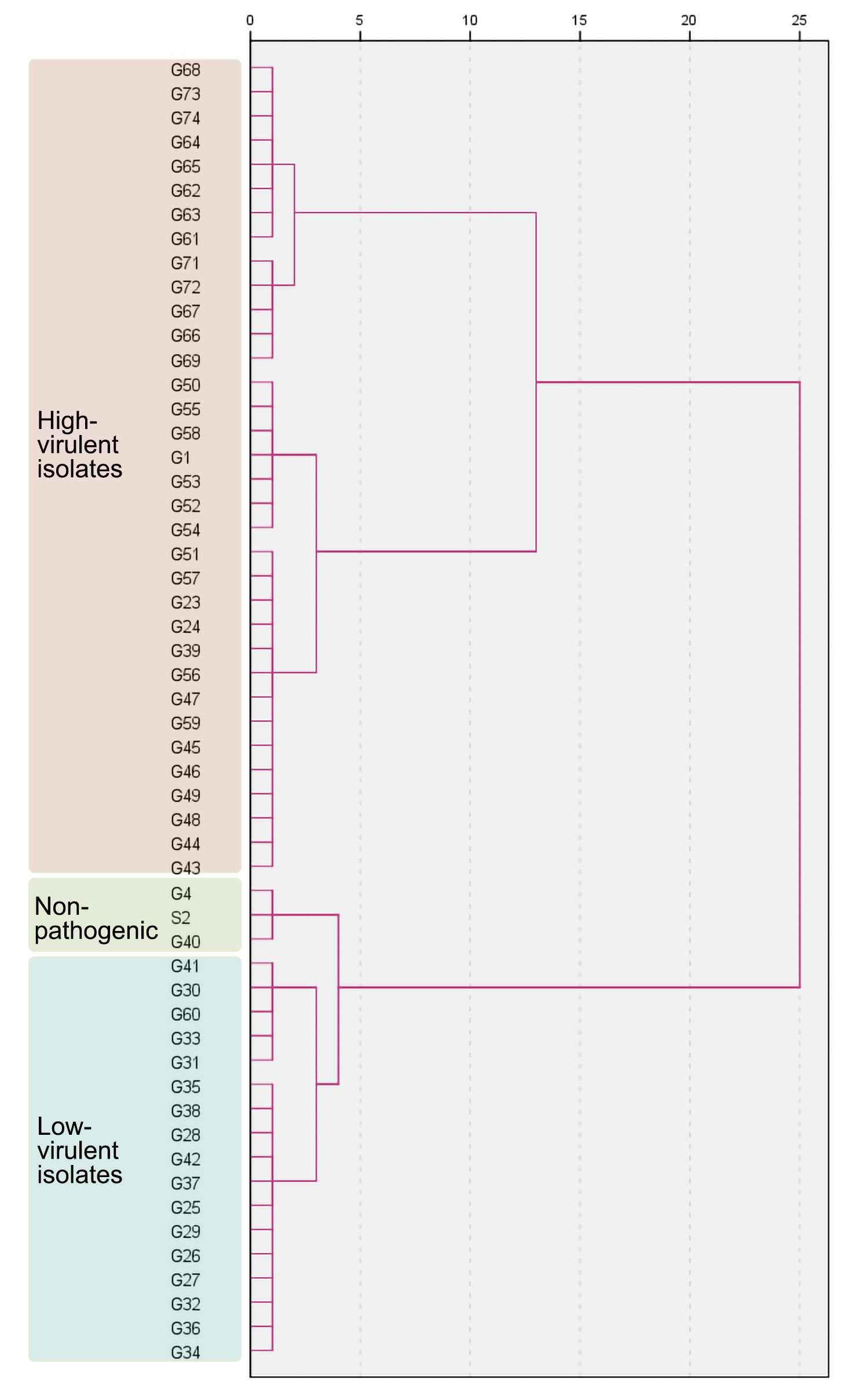

Supplement: Supplementary Figure 1 — Hierarchical cluster analysis of pathogenicity data with the Ward’s method in SPSS 19.0. [file Image_1.TIF]
