# Supplementary material for: Genetic Causes of Non-pathogenic Pseudomonas syringae pv. actinidiae Isolates in Kiwifruit Orchards
Source: Front Microbiol. 2021 Mar 25;12:650099. doi: 10.3389/fmicb.2021.650099 (PMC8027508; doi:10.3389/fmicb.2021.650099)
Supplement: Supplementary Table 1 — Primers used in this study. [file Table_1.DOCX]

TABLE S1 Primers used in this study

| **No.** | **Primer pairs** | **Primer sequence (5' - 3')** | **Product size (bp)** | **Restriction site** | **Reference** |
| --- | --- | --- | --- | --- | --- |
| 1 | PsaF | cagaggcgctaacgaggaaa | 311 |  | Balestra et al. 2013 |
|  | PsaR | tcgattttgccgtgatgagtc |  |  |  |
| 2 | P0F | ctgcaacaggcgacggcgaggc | 243 |  | Gallelli et al. 2014) |
|  | P6R | cataggcttctggttttcttcctgatcc |  |  |  |
| 3 | TR2-F | tgaaagaactgtgccaatttgtg | 653 |  | Zhao et al. 2019b |
|  | TR2-R | ttctggtagtttacaccgcctc |  |  |  |
| 4 | TR5-F | gtagcgtaggaacgatattcaagtt | 653 |  | Zhao et al. 2019b |
|  | TR5-R | gcgcaaggtttacgggttt |  |  |  |
| 5 | TR7-F | ggtacgcattccaatcaacc | 191-219 |  | Zhao et al. 2019b |
|  | TR7-R | aagggcaaatgatcgctaact |  |  |  |
| 6 | TR8-F | gtgcgttaagtatgtagcgtctt | 129-136 |  | Zhao et al. 2019b |
|  | TR8-R | cgctggagttgcgaagg |  |  |  |
| 7 | TR14-F | gattggtgacgttgcgatga | 61-101 |  | Zhao et al. 2019b |
|  | TR14-R | ttgttgccctacacgctcta |  |  |  |
| 8 | TR16-F | gcctgaaccgtccgtgg | 170-193 |  | Zhao et al. 2019b |
|  | TR16-R | cgacacccagttcattacgaat |  |  |  |
| 9 | TR17-F | aatctgcacctcgccgactc | 145-157 |  | Zhao et al. 2019b |
|  | TR17-R | caagaaggtcaacccgtccc |  |  |  |
| 10 | TR19-F | catgcgggcaatctgatagt | 174-202 |  | Zhao et al. 2019b |
|  | TR19-R | caagcaggagatggaagagc |  |  |  |
| 11 | TR22-F | ctgcaccgaagcgatgacc | 161-170 |  | Zhao et al. 2019b |
|  | TR22-R | cgccaacattgccctgcta |  |  |  |
| 12 | TR23-F | aagtcggcgagcgaagataa | 197-257 |  | Zhao et al. 2019b |
|  | TR23-R | gttgcacgatagcacaacctct |  |  |  |
| 13 | hrpRN-F | gccaagcttgcattggagtaacgatagattaaa | 873 | *HindIII* | This study |
|  | hrpRN-R | taatcatggtccagtcgtggctgagcag |  |  |  |
| 14 | gfpF | tgttgcccggactgcaattttagagagacgg | 841 |  | This study |
|  | gfpR | ccacgactggaccatgattacgccaagct |  |  |  |
| 15 | hrpRC-F | aattgcagtccgggcaacatccgtgaa | 784 |  | This study |
|  | hrpRC-R | tacgaattcgtcgtccagcgagcatt |  | *EcoRI* |  |
| 16 | hrpR_RTF | gtgaacagatcctgccattg | wildtype: 171 Insertion mutant: 1012 |  | This study |
|  | hrpR_RTR | ggaagccgagtacgaagc |  |  |  |
| 17 | SacB-F | gcaaacactggaactgaagatgg | 478 |  | Zhao et al. 2019b |
|  | SacB-R | ttcctttcgcttgaggtacagc |  |  |  |
| 18 | gyrA-RT-F | aacattccgccgcataacc | 234 |  | Zhao et al. 2019b |
|  | gyrA-RT-R | ctgacgaccgcccacctt |  |  |  |
| 19 | gyrB-RT-F | acccgaacgaagccaaagc | 201 |  | Zhao et al. 2019b |
|  | gyrB-RT-R | atccgccagcagagtccc |  |  |  |
| 20 | hrpL-RT-F | attgctgccgacccacatc | 130 |  | Zhao et al. 2019b |
|  | hrpL-RT-R | acatacccgcttcgtctacctg |  |  |  |
| 21 | hopBB1-1-F | cgcgagctcatgggtaatatttgtggtacttcc | 843 | *SacI* | This study |
|  | hopBB1-1-R | cgcggatcctcattcatcagaccgagaatagttgt |  | *BamHI* |  |
| 22 | hopBB1-2-F | cgcgagctcatgggcaatatttgtggtacttcc | 843 | *SacI* | This study |
|  | hopBB1-2-R | cgcggatcctatccatcagaccgagaatagttg |  | *BamHI* |  |
| 23 | hopBB1-1-E_F | ggaattccatatgggtaatatttgtggtacttcc | 861 | *NdeI* | This study |
|  | hopBB1-1-E_R | aactgcagttattcatcagaccgagaatagttg |  | *PstI* |  |
| 24 | hopBB1-2-E_F | ggaattccatatgggcaatatttgtggtacttc | 863 | *NdeI* | This study |
|  | hopBB1-2-E_R | aactgcaggctatccatcagaccgagaat |  | *PstI* |  |
