# Supplementary material for: Genetic Causes of Non-pathogenic Pseudomonas syringae pv. actinidiae Isolates in Kiwifruit Orchards
Source: Front Microbiol. 2021 Mar 25;12:650099. doi: 10.3389/fmicb.2021.650099 (PMC8027508; doi:10.3389/fmicb.2021.650099)
Supplement: Supplementary Table 2 — The detailed information of Psa3 isolates and MLVA typing results. [file Table_2.docx]

TABLE S2 The detailed information of Psa3 isolates and MLVA typing results

| **Strain No.** | **Geographical source** | **Sample No.** | **Host Tissue** | **Year** | **MLVA Typing** | **TR2** | **TR22** | **TR5** | **TR8** | **TR14** | **TR23** | **TR17** | **TR19** | **TR7** | **TR16** |
| --- | --- | --- | --- | --- | --- | --- | --- | --- | --- | --- | --- | --- | --- | --- | --- |
| S23 | Shaanxi | 201703-1 | trunk | 2017 | Clade 2 | 8 | 2 | 3 | 9 | 3 | 4 | 2 | 4 | 2 | 1 |
| S29 | Shaanxi | 201703-2 | trunk | 2017 | Clade 3 | 4 | 2 | 4 | 5 | 4 | 9 | 2 | 4 | 3 | 2 |
| S3 | Shaanxi | 201703-3 | trunk | 2017 | Clade 1 | 6 | 3 | 4 | 4 | 5 | 11 | 3 | 7 | 4 | 2 |
| S5 | Shaanxi | 201703-4 | trunk | 2017 | Clade 1 | 6 | 3 | 4 | 4 | 5 | 11 | 3 | 8 | 5 | 2 |
| G1 | Xifeng | 20170415-1 | trunk | 2017 | Clade 4 | 4 | 2 | 3 | 5 | 6 | 4 | 2 | 4 | 7 | 2 |
| G4 | Xifeng | 20170415-1 | trunk | 2017 | Clade 4 | 4 | 2 | 3 | 5 | 6 | 4 | 2 | 4 | 7 | 2 |
| G5 | Xifeng | 20170415-1 | trunk | 2017 | Clade 4 | 4 | 2 | 3 | 5 | 6 | 4 | 2 | 4 | 7 | 2 |
| G7 | Xifeng | 20170415-2 | trunk | 2017 | Clade 4 | 4 | 2 | 3 | 5 | 6 | 4 | 2 | 4 | 7 | 2 |
| G10 | Xifeng | 20170415-2 | trunk | 2017 | Clade 4 | 4 | 2 | 3 | 5 | 6 | 4 | 2 | 4 | 7 | 2 |
| G12 | Xifeng | 20170415-2 | trunk | 2017 | Clade 4 | 4 | 2 | 3 | 5 | 6 | 4 | 2 | 4 | 7 | 2 |
| G14 | Xifeng | 20170415-2 | trunk | 2017 | Clade 4 | 4 | 2 | 3 | 5 | 6 | 4 | 2 | 4 | 7 | 2 |
| G16 | Xifeng | 20170415-3 | trunk | 2017 | Clade 4 | 4 | 2 | 3 | 5 | 5 | 4 | 2 | 4 | 6 | 2 |
| G18 | Xifeng | 20170415-3 | trunk | 2017 | Clade 4 | 4 | 2 | 3 | 5 | 5 | 4 | 2 | 4 | 6 | 2 |
| G20 | Xifeng | 20170415-3 | trunk | 2017 | Clade 4 | 4 | 2 | 3 | 5 | 5 | 4 | 2 | 4 | 6 | 2 |
| G23 | Xiuwen | 20170422-1 | trunk | 2017 | Clade 4 | 4 | 2 | 3 | 9 | 5 | 4 | 2 | 4 | 6 | 2 |
| G24 | Xiuwen | 20170422-1 | trunk | 2017 | Clade 4 | 4 | 2 | 3 | 3 | 5 | 4 | 2 | 4 | 6 | 2 |
| G37 | Xiuwen | 20170422-10 | trunk | 2017 | Clade 4 | 4 | 2 | 3 | 4 | 5 | 4 | 2 | 4 | 7 | 2 |
| G38 | Xiuwen | 20170422-11 | trunk | 2017 | Clade 4 | 4 | 2 | 3 | 4 | 5 | 4 | 2 | 4 | 6 | 2 |
| G39 | Xiuwen | 20170422-11 | trunk | 2017 | Clade 4 | 4 | 2 | 3 | 4 | 5 | 4 | 2 | 4 | 6 | 2 |
| G40 | Xiuwen | 20170422-12 | leaf | 2017 | Clade 4 | 4 | 2 | 3 | 5 | 6 | 4 | 2 | 5 | 6 | 2 |
| G41 | Xiuwen | 20170422-12 | leaf | 2017 | Clade 4 | 4 | 2 | 3 | 5 | 6 | 4 | 2 | 5 | 6 | 2 |
| G42 | Xiuwen | 20170422-13 | leaf | 2017 | Clade 4 | 4 | 2 | 3 | 5 | 6 | 4 | 2 | 5 | 6 | 2 |
| G43 | Xiuwen | 20170422-14 | leaf | 2017 | Clade 4 | 4 | 2 | 3 | 5 | 6 | 4 | 2 | 5 | 6 | 2 |
| G44 | Xiuwen | 20170422-14 | leaf | 2017 | Clade 4 | 4 | 2 | 3 | 5 | 6 | 4 | 2 | 5 | 6 | 2 |
| G45 | Xiuwen | 20170422-14 | leaf | 2017 | Clade 4 | 4 | 2 | 3 | 5 | 6 | 4 | 2 | 5 | 6 | 2 |
| G46 | Xiuwen | 20170422-15 | leaf | 2017 | Clade 4 | 6 | 2 | 3 | 5 | 5 | 4 | 2 | 4 | 7 | 2 |
| G47 | Xiuwen | 20170422-15 | leaf | 2017 | Clade 4 | 4 | 2 | 3 | 5 | 5 | 4 | 2 | 4 | 7 | 2 |
| G48 | Xiuwen | 20170422-16 | leaf | 2017 | Clade 3 | 4 | 2 | 4 | 7 | 4 | 10 | 2 | 4 | 5 | 2 |
| G49 | Xiuwen | 20170422-16 | leaf | 2017 | Clade 3 | 4 | 2 | 4 | 7 | 4 | 10 | 2 | 4 | 5 | 2 |
| G50 | Xiuwen | 20170422-17 | leaf | 2017 | Clade 3 | 4 | 2 | 4 | 7 | 4 | 10 | 2 | 4 | 5 | 2 |
| G51 | Xiuwen | 20170422-17 | leaf | 2017 | Clade 3 | 4 | 2 | 4 | 7 | 4 | 10 | 2 | 4 | 5 | 2 |
| G25 | Xiuwen | 20170422-2 | root | 2017 | Clade 1 | 6 | 3 | 4 | 4 | 5 | 15 | 3 | 8 | 4 | 2 |
| G26 | Xiuwen | 20170422-3 | trunk | 2017 | Clade 4 | 4 | 2 | 3 | 3 | 5 | 4 | 2 | 4 | 6 | 2 |
| G27 | Xiuwen | 20170422-5 | leaf | 2017 | Clade 4 | 4 | 2 | 3 | 3 | 5 | 4 | 2 | 4 | 6 | 2 |
| G28 | Xiuwen | 20170422-5 | leaf | 2017 | Clade 4 | 4 | 2 | 3 | 3 | 5 | 4 | 2 | 4 | 6 | 2 |
| G29 | Xiuwen | 20170422-6 | leaf | 2017 | Clade 4 | 4 | 2 | 4 | 3 | 5 | 4 | 2 | 4 | 6 | 2 |
| G30 | Xiuwen | 20170422-6 | leaf | 2017 | Clade 4 | 4 | 2 | 3 | 3 | 5 | 4 | 2 | 4 | 6 | 2 |
| G31 | Xiuwen | 20170422-7 | trunk | 2017 | Clade 4 | 4 | 2 | 3 | 3 | 5 | 4 | 2 | 4 | 6 | 2 |
| G32 | Xiuwen | 20170422-7 | trunk | 2017 | Clade 4 | 4 | 2 | 3 | 3 | 5 | 4 | 2 | 4 | 6 | 2 |
| G33 | Xiuwen | 20170422-8 | leaf | 2017 | Clade 4 | 4 | 2 | 3 | 3 | 5 | 4 | 2 | 4 | 6 | 2 |
| G34 | Xiuwen | 20170422-8 | leaf | 2017 | Clade 4 | 4 | 2 | 3 | 4 | 6 | 4 | 2 | 5 | 6 | 2 |
| G35 | Xiuwen | 20170422-9 | trunk | 2017 | Clade 4 | 4 | 2 | 3 | 4 | 5 | 4 | 2 | 4 | 6 | 2 |
| G36 | Xiuwen | 20170422-9 | trunk | 2017 | Clade 4 | 4 | 2 | 3 | 4 | 5 | 4 | 2 | 4 | 6 | 2 |
| G52 | Xiuwen | 20170528-1 | leaf | 2017 | Clade 1 | 6 | 3 | 4 | 4 | 5 | 11 | 3 | 8 | 4 | 2 |
| G53 | Xiuwen | 20170528-1 | leaf | 2017 | Clade 1 | 6 | 3 | 4 | 4 | 5 | 11 | 3 | 8 | 4 | 2 |
| G63 | Xiuwen | 20170528-11 | leaf | 2017 | Clade 3 | 4 | 2 | 4 | 7 | 4 | 11 | 2 | 4 | 5 | 2 |
| G64 | Xiuwen | 20170528-11 | leaf | 2017 | Clade 3 | 6 | 2 | 4 | 7 | 4 | 11 | 2 | 4 | 5 | 2 |
| G65 | Xiuwen | 20170528-12 | leaf | 2017 | Clade 3 | 6 | 2 | 4 | 7 | 4 | 11 | 2 | 4 | 5 | 2 |
| G66 | Xiuwen | 20170528-13 | leaf | 2017 | Clade 4 | 4 | 2 | 3 | 5 | 6 | 4 | 2 | 5 | 6 | 2 |
| G67 | Xiuwen | 20170528-14 | leaf | 2017 | Clade 4 | 6 | 2 | 3 | 5 | 6 | 4 | 2 | 4 | 6 | 2 |
| G68 | Xiuwen | 20170528-15 | leaf | 2017 | Clade 3 | 4 | 2 | 4 | 7 | 4 | 10 | 2 | 4 | 5 | 2 |
| G69 | Xiuwen | 20170528-15 | leaf | 2017 | Clade 3 | 4 | 2 | 4 | 7 | 4 | 10 | 2 | 4 | 5 | 2 |
| G70 | Xiuwen | 20170528-16 | leaf | 2017 | Clade 3 | 4 | 2 | 4 | 7 | 4 | 10 | 2 | 4 | 5 | 2 |
| G71 | Xiuwen | 20170528-16 | leaf | 2017 | Clade 3 | 4 | 2 | 4 | 7 | 4 | 10 | 2 | 4 | 5 | 2 |
| G72 | Xiuwen | 20170528-16 | leaf | 2017 | Clade 3 | 4 | 2 | 4 | 7 | 4 | 10 | 2 | 4 | 5 | 2 |
| G73 | Xiuwen | 20170528-17 | leaf | 2017 | Clade 1 | 6 | 3 | 4 | 4 | 5 | 11 | 3 | 8 | 4 | 2 |
| G74 | Xiuwen | 20170528-17 | leaf | 2017 | Clade 1 | 6 | 3 | 4 | 4 | 5 | 11 | 3 | 8 | 4 | 2 |
| G54 | Xiuwen | 20170528-2 | leaf | 2017 | Clade 1 | 6 | 3 | 4 | 4 | 5 | 11 | 3 | 8 | 4 | 2 |
| G55 | Xiuwen | 20170528-2 | leaf | 2017 | Clade 1 | 6 | 3 | 4 | 4 | 5 | 11 | 3 | 8 | 4 | 2 |
| G56 | Xiuwen | 20170528-2 | leaf | 2017 | Clade 1 | 6 | 3 | 4 | 4 | 5 | 11 | 3 | 8 | 4 | 2 |
| G57 | Xiuwen | 20170528-3 | leaf | 2017 | Clade 1 | 6 | 3 | 4 | 4 | 5 | 11 | 3 | 8 | 4 | 2 |
| G58 | Xiuwen | 20170528-3 | leaf | 2017 | Clade 1 | 6 | 3 | 4 | 4 | 6 | 11 | 3 | 8 | 4 | 2 |
| G59 | Xiuwen | 20170528-4 | leaf | 2017 | Clade 1 | 6 | 3 | 4 | 4 | 5 | 11 | 3 | 8 | 4 | 2 |
| G60 | Xiuwen | 20170528-5 | leaf | 2017 | Clade 1 | 6 | 3 | 4 | 4 | 5 | 11 | 3 | 8 | 4 | 2 |
| G61 | Xiuwen | 20170528-8 | leaf | 2017 | Clade 1 | 6 | 3 | 4 | 4 | 5 | 10 | 3 | 8 | 4 | 2 |
| G62 | Xiuwen | 20170528-8 | leaf | 2017 | Clade 1 | 6 | 3 | 4 | 4 | 5 | 10 | 3 | 8 | 4 | 2 |
| S21 | Shaanxi | 201707-3 | trunk | 2017 | Clade 1 | 6 | 3 | 4 | 5 | 4 | 11 | 3 | 8 | 3 | 2 |
| S2 | Shaanxi | 201707-4 | trunk | 2017 | Clade 1 | 6 | 3 | 4 | 4 | 5 | 11 | 3 | 8 | 4 | 2 |
| S14 | Shaanxi | 201707-5 | trunk | 2017 | Clade 1 | 6 | 3 | 4 | 4 | 5 | 11 | 3 | 8 | 4 | 2 |
| S26 | Shaanxi | 201712-4 | trunk | 2017 | Clade 2 | 8 | 2 | 3 | 9 | 3 | 4 | 2 | 4 | 2 | 1 |
| S30 | Shaanxi | 201712-5 | trunk | 2017 | Clade 2 | 8 | 2 | 3 | 9 | 3 | 3 | 2 | 4 | 2 | 1 |
| GM50-1 | Liupanshui | 20180321-6 | trunk | 2018 | Clade 1-2 | 6 | 3 | 3 | 3 | 6 | 10 | 3 | 7 | 4 | 2 |
| GM50-2 | Liupanshui | 20180321-6 | trunk | 2018 | Clade 1-2 | 6 | 3 | 3 | 3 | 6 | 9 | 3 | 7 | 4 | 2 |
| GM51-1 | Liupanshui | 20180321-7 | trunk | 2018 | Clade 1-2 | 6 | 3 | 3 | 3 | 6 | 9 | 3 | 7 | 4 | 2 |
| GM51-2 | Liupanshui | 20180321-7 | trunk | 2018 | Clade 1-2 | 6 | 3 | 3 | 3 | 6 | 9 | 3 | 7 | 4 | 2 |
| GM52 | Liupanshui | 20180321-8 | trunk | 2018 | Clade 1-2 | 6 | 3 | 3 | 3 | 6 | 9 | 3 | 7 | 4 | 2 |
| GM53 | Liupanshui | 20180321-9 | trunk | 2018 | Clade 4 | 4 | 2 | 3 | 3 | 6 | 3 | 2 | 4 | 6 | 2 |
| K10 | Liupanshui | 20200306-10 | flower | 2020 | Clade 1-2 | 6 | 3 | 3 | 3 | 6 | 9 | 3 | 7 | 4 | 2 |
| K15 | Xifeng | 20200306-15 | trunk | 2020 | Clade 5 | 6 | 2 | 3 | 4 | 6 | 6 | 2 | 4 | 7 | 2 |
| K20-2 | Liupanshui | 20200306-20 | trunk | 2020 | Clade 5 | 6 | 2 | 3 | 4 | 6 | 6 | 2 | 4 | 7 | 2 |
| K6 | Liupanshui | 20200306-6 | trunk | 2020 | Clade 4 | 4 | 2 | 3 | 3 | 6 | 2 | 2 | 4 | 6 | 2 |
| K8 | Liupanshui | 20200306-8 | trunk | 2020 | Clade 1-2 | 6 | 3 | 3 | 3 | 6 | 10 | 2 | 7 | 4 | 2 |
| K9 | Liupanshui | 20200306-9 | trunk | 2020 | Clade 1-2 | 6 | 3 | 3 | 4 | 6 | 10 | 3 | 7 | 4 | 2 |
| K47-1 | Xifeng | 20200419-47 | trunk | 2020 | Clade 8 | 4 | 2 | 3 | 7 | 4 | 15 | 2 | 7 | 9 | 2 |
| K47-2 | Xifeng | 20200419-47 | trunk | 2020 | Clade 8 | 4 | 2 | 3 | 7 | 5 | 15 | 3 | 7 | 9 | 2 |
| K48-2 | Xifeng | 20200419-48 | leaf | 2020 | Clade 1-2 | 6 | 3 | 3 | 3 | 6 | 10 | 3 | 7 | 4 | 2 |
| K49 | Xifeng | 20200419-49 | leaf | 2020 | Clade 1-2 | 6 | 3 | 3 | 3 | 6 | 9 | 3 | 7 | 4 | 2 |
| K50-1 | Xifeng | 20200419-50 | leaf | 2020 | Clade 1-2 | 6 | 3 | 3 | 3 | 6 | 9 | 3 | 7 | 4 | 2 |
| K50-2 | Xifeng | 20200419-50 | leaf | 2020 | Clade 1-2 | 6 | 3 | 3 | 3 | 5 | 10 | 3 | 7 | 4 | 2 |
| K52 | Xifeng | 20200419-52 | leaf | 2020 | Clade 1-2 | 6 | 3 | 3 | 3 | 5 | 10 | 3 | 7 | 9 | 2 |
| K53-1 | Xifeng | 20200419-53 | flower | 2020 | Clade 1-2 | 6 | 3 | 3 | 3 | 6 | 10 | 3 | 7 | 4 | 2 |
| K54-1 | Xifeng | 20200419-54 | flower | 2020 | Clade 1-2 | 6 | 3 | 3 | 3 | 6 | 10 | 3 | 7 | 4 | 2 |
| K54-2 | Xifeng | 20200419-54 | flower | 2020 | Clade 1-2 | 6 | 3 | 3 | 3 | 6 | 10 | 3 | 7 | 4 | 2 |
| K55-1 | Xifeng | 20200419-55 | flower | 2020 | Clade 1-2 | 6 | 3 | 3 | 3 | 6 | 10 | 3 | 7 | 4 | 2 |
| K55-2 | Xifeng | 20200419-55 | flower | 2020 | Clade 1-2 | 6 | 3 | 3 | 3 | 6 | 10 | 3 | 7 | 4 | 2 |
| K56-1 | Xifeng | 20200419-56 | flower | 2020 | Clade 1-2 | 6 | 3 | 3 | 3 | 6 | 10 | 3 | 7 | 4 | 2 |
| K56-2 | Xifeng | 20200419-56 | flower | 2020 | Clade 1-2 | 6 | 3 | 3 | 3 | 6 | 10 | 3 | 7 | 4 | 2 |
| K57-1 | Xifeng | 20200419-57 | flower | 2020 | Clade 1-2 | 6 | 3 | 3 | 3 | 6 | 10 | 3 | 7 | 4 | 2 |
| K57-2 | Xifeng | 20200419-57 | flower | 2020 | Clade 1-2 | 6 | 3 | 3 | 3 | 6 | 10 | 3 | 7 | 4 | 2 |
| K58-1 | Xifeng | 20200419-58 | flower | 2020 | Clade 8 | 4 | 2 | 3 | 7 | 5 | 15 | 3 | 7 | 9 | 2 |
| K58-2 | Xifeng | 20200419-58 | flower | 2020 | Clade 1-2 | 6 | 3 | 3 | 3 | 6 | 10 | 3 | 7 | 4 | 1 |
| K59-2 | Xifeng | 20200419-59 | flower | 2020 | Clade 1-2 | 6 | 3 | 3 | 3 | 6 | 10 | 3 | 7 | 4 | 1 |
| K60-1 | Xifeng | 20200419-60 | flower | 2020 | Clade 1-2 | 6 | 3 | 3 | 3 | 6 | 10 | 2 | 7 | 4 | 2 |
| K60-2 | Xifeng | 20200419-60 | flower | 2020 | Clade 1-2 | 6 | 3 | 3 | 3 | 6 | 10 | 3 | 7 | 4 | 1 |
| K62-1 | Xifeng | 20200419-62 | flower | 2020 | Clade 1-2 | 6 | 3 | 3 | 3 | 6 | 10 | 2 | 7 | 4 | 2 |
| K62-2 | Xifeng | 20200419-62 | flower | 2020 | Clade 1-2 | 6 | 3 | 3 | 3 | 6 | 10 | 3 | 7 | 4 | 2 |
| K64-1 | Xifeng | 20200419-64 | trunk | 2020 | Clade 1-2 | 6 | 3 | 3 | 3 | 6 | 10 | 2 | 7 | 4 | 2 |
| K64-2 | Xifeng | 20200419-64 | trunk | 2020 | Clade 1-2 | 6 | 3 | 3 | 3 | 6 | 10 | 3 | 7 | 4 | 2 |
| K66-1 | Xifeng | 20200419-66 | trunk | 2020 | Clade 1-2 | 6 | 3 | 3 | 3 | 6 | 10 | 3 | 7 | 4 | 2 |
| K66-2 | Xifeng | 20200419-66 | trunk | 2020 | Clade 1-2 | 6 | 3 | 3 | 3 | 6 | 10 | 2 | 7 | 4 | 2 |
| GHY01 | Liupanshui | 202005-2-3 | leaf | 2020 | Clade 1-2 | 6 | 3 | 3 | 3 | 6 | 9 | 3 | 7 | 4 | 2 |
| GHY02 | Liupanshui | 202005-2-3 | leaf | 2020 | Clade 1-2 | 6 | 3 | 3 | 3 | 6 | 9 | 3 | 7 | 4 | 2 |
| GHY03 | Liupanshui | 202005-2-4 | trunk | 2020 | Clade 1-2 | 6 | 3 | 3 | 3 | 6 | 9 | 3 | 7 | 4 | 2 |
| GHY04 | Liupanshui | 202005-2-4 | trunk | 2020 | Clade 1-2 | 6 | 3 | 3 | 3 | 6 | 9 | 3 | 7 | 4 | 2 |
| GHY05 | Liupanshui | 202005-2-5 | trunk | 2020 | Clade 1-2 | 6 | 3 | 3 | 3 | 6 | 9 | 3 | 7 | 4 | 2 |
| GHY06 | Liupanshui | 202005-2-5 | trunk | 2020 | Clade 1-2 | 6 | 3 | 3 | 3 | 6 | 9 | 3 | 7 | 4 | 2 |
| GHY07 | Liupanshui | 202005-3-1 | trunk | 2020 | Clade 8 | 4 | 2 | 3 | 7 | 5 | 15 | 2 | 7 | 9 | 2 |
| GHY10 | Liupanshui | 202005-3-2 | trunk | 2020 | Clade 8 | 4 | 2 | 3 | 7 | 5 | 15 | 2 | 7 | 9 | 2 |
| GHY15 | Liupanshui | 202005-3-5 | trunk | 2020 | Clade 8 | 4 | 2 | 3 | 7 | 5 | 15 | 2 | 7 | 9 | 2 |
| GHY16 | Liupanshui | 202005-3-5 | trunk | 2020 | Clade 8 | 4 | 2 | 3 | 7 | 5 | 15 | 2 | 7 | 9 | 2 |
| GHY19 | Liupanshui | 202005-3-7 | trunk | 2020 | Clade 8 | 4 | 2 | 3 | 7 | 5 | 15 | 2 | 7 | 9 | 2 |
| GHY20 | Liupanshui | 202005-3-7 | trunk | 2020 | Clade 8 | 4 | 2 | 3 | 7 | 5 | 15 | 2 | 7 | 9 | 2 |

**Note:** Shaanxi indicates Shaanxi Province, China; Liupanshui indicates Liupanshui City, Guizhou Province, China; Xifeng and Xiuwen are in Guiyang City, Guizhou Province, China
